# Rho kinase inhibitor enables cell-based therapy for corneal endothelial dysfunction

**DOI:** 10.1038/srep26113

**Published:** 2016-05-18

**Authors:** Naoki Okumura, Yuji Sakamoto, Keita Fujii, Junji Kitano, Shinichiro Nakano, Yuki Tsujimoto, Shin-ichiro Nakamura, Morio Ueno, Michio Hagiya, Junji Hamuro, Akifumi Matsuyama, Shingo Suzuki, Takashi Shiina, Shigeru Kinoshita, Noriko Koizumi

**Affiliations:** 1 Department of Biomedical Engineering, Faculty of Life and Medical Sciences, Doshisha University, Kyotanabe, Japan; 2 Research Laboratory, Senju Pharmaceutical Co., Ltd., Kobe, Japan; 3 Research Center of Animal Life Science, Shiga University of Medical Science, Otsu, Japan; 4 Department of Ophthalmology, Kyoto Prefectural University of Medicine, Kyoto, Japan; 5 Platform of Therapeutics for Rare Disease, National Institutes of Biomedical Innovation, Health and Nutrition, Osaka, Japan; 6 Department of Molecular Life Science, Division of Basic Medical Science and Molecular Medicine, Tokai University School of Medicine, Isehara, Japan; 7 Department of Frontier Medical Science and Technology for Ophthalmology, Kyoto Prefectural University of Medicine, Kyoto, Japan

## Abstract

The corneal endothelium maintains corneal transparency; consequently, its dysfunction causes severe vision loss. Tissue engineering-based therapy, as an alternative to conventional donor corneal transplantation, is anticipated to provide a less invasive and more effective therapeutic modality. We conducted a preclinical study for cell-based therapy in a primate model and demonstrated regeneration of the corneal endothelium following injection of cultured monkey corneal endothelial cells (MCECs) or human CECs (HCECs), in combination with a Rho kinase (ROCK) inhibitor, Y-27632, into the anterior chamber. We also evaluated the safety and efficacy of Good Manufacturing Practice (GMP)-grade HCECs, similar to those planned for use as transplant material for human patients in a clinical trial, and we showed that the corneal endothelium was regenerated without adverse effect. We also showed that CEC engraftment is impaired by limited substrate adhesion, which is due to actomyosin contraction induced by dissociation-induced activation of ROCK/MLC signaling. Inclusion of a ROCK inhibitor improves efficiency of engraftment of CECs and enables cell-based therapy for treating corneal endothelial dysfunction as a clinically relevant therapy.

The corneal endothelium maintains corneal transparency by a pump and barrier function that reduces the aqueous humor flow into corneal stroma. Consequently, endothelial dysfunction causes severe vision loss. Any damage to the corneal endothelium due to pathological status, such as Fuchs endothelial corneal dystrophies and surgical trauma, is compensated by migration and spreading of the remaining corneal endothelial cells (CECs)^1^. However, once the cell density (2500–3000 cells/mm^2^ in healthy individuals) drops lower than a critical level (<1000 cells/mm^2^), decompensation of endothelial function induces corneal haziness^2^.

Corneal transplantation is the only therapeutic choice for treating corneal endothelial dysfunction, but is hampered by a shortage of donor corneas, the difficulty of the surgical procedure, and graft failure in acute and chronic phases. Therefore, researchers are actively seeking to develop tissue engineering based therapeutics^3^,^4^. For instance, some investigators, including us, have cultured CECs on scaffolds in the form of a sheet, and have shown in animal models that corneal endothelial dysfunction can be treated by sheet transplantation^5^,^6^,^7^. In addition to sheet transplantation, we have demonstrated that a Rho kinase (ROCK) inhibitor, Y-27632, improves the engraftment of transplanted CECs and that injection of CECs in the form of a cell suspension can regenerate the corneal endothelium^8^.

This paper reports a preclinical study for corneal endothelial cell-based therapy conducted in a cynomolgus monkey model. Corneal endothelium was regenerated by injection of cultured monkey CECs (MCECs) and human CECs (HCECs), in combination with the ROCK inhibitor, and the regeneration occurred without adverse effects, such as rejection, secondary glaucoma, or aberrant ectopic cell transplantation. We also showed that CEC engraftment is impaired by actomyosin contraction induced by cell dissociation through activation of Rho/ROCK/MLC signaling. Inclusion of a ROCK inhibitor enhances the adhesion to the extracellular matrix (ECM) by counteracting this cascade. Taken together, the results from this preclinical study in a primate model demonstrate that ROCK inhibitors enhance cell engraftment, thereby enabling CEC injection as a clinically relevant cell-based therapy for treating corneal endothelial dysfunction.

## Results

### Cultivated MCEC injection in combination with a ROCK inhibitor in a monkey model

**Figure 1:**
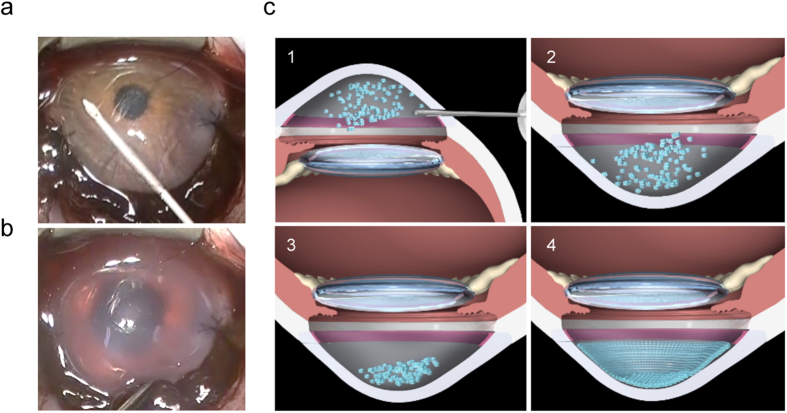
Cultured corneal endothelial cell (CEC) injection in the corneal endothelial dysfunction model. (**a**) To create monkey corneal endothelial dysfunction models, the corneal endothelium was completely scraped from the Descemet’s membrane with a 20-gauge silicone needle. CECs (5.0 × 10^5^ cells), suspended in 200 μl of DMEM supplemented with 100 μM of Y-27632 (a ROCK inhibitor), were injected into the anterior chamber with a 26-gauge needle. (**b**) After confirmation of the absence of leakage of the injected CECs, the eyes were kept in the face-down position for 3 hours with the monkeys under general anesthesia. (**c**) Schematic images show the cultured CEC injection procedure. (1) injection of cultured CECs with ROCK inhibitor into the anterior chamber, (2) face-down position for CECs to sink down to the anterior chamber side of the cornea, (3) animal is maintained in the face-down position for 3 hours, (4) regeneration of corneal endothelium by injected cultured CECs.

**Figure 2:**
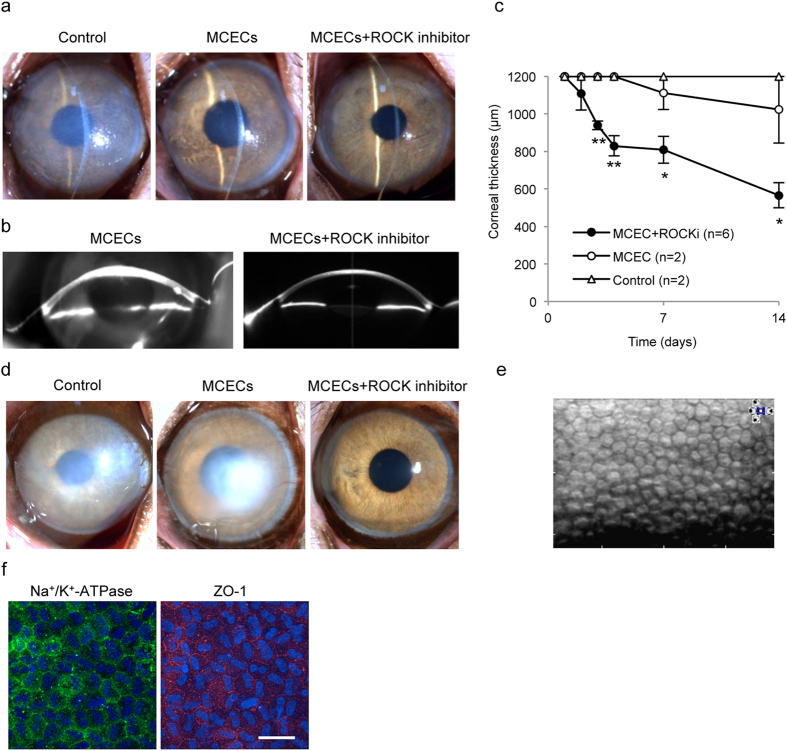
Preclinical research of cultured monkey corneal endothelial cell (MCEC) injection in combination with a ROCK inhibitor in a monkey corneal endothelial dysfunction model. (**a**) A representative slit-lamp image shows the monkey corneal endothelial dysfunction model (left) (n = 2). A representative slit-lamp image shows the corneal endothelial dysfunction model following injection of MCECs (5.0 × 10^5^ cells) suspended in 200 μl of DMEM without (middle) (n = 2) or with (right) (n = 6) the ROCK inhibitor Y-27632. All images were obtained 14 days after treatment. (**b**) Scheimpflug images were obtained from monkeys injected with MCECs without or with Y-27632, obtained with a Pentacam^®^ instrument at 14 days after cell injection. (**c**) The mean central corneal thickness was evaluated by ultrasound pachymetry. *P < 0.01, **P < 0.05. (**d**) Representative slit-lamp images were obtained from monkeys injected with MCECs without or with Y-27632. Images were obtained 1 year after injection (n = 2). (**e**) Regenerated corneal endothelium was evaluated by non-contact specular microscopy 1 year after treatment in a monkey injected MCECs with the ROCK inhibitor Y-27632. Note that no image was obtained for the monkey injected with MCECs without Y-27632. (**f**) Immunostaining of function-related markers was determined in CECs (Na^+^/K^+^-ATPase and ZO-1) in regenerated corneal endothelium. Nuclei were stained with DAPI. Scale bar: 100 μm.

We completely removed the corneal endothelium to generate the corneal endothelial dysfunction model. We then injected cultured MCECs (5.0 × 10^5^ cells) into the anterior chamber using a 26G needle and confirmed the absence of leakage of injected cells from the wound (Fig. 1a,b). The schematic images in Fig. 1c show the surgical procedure: (1) cultured CEC injection in combination with the ROCK inhibitor into the anterior chamber, (2) face-down position to allow CECs to sink to the Descemet’s membrane, (3) the face-down position is maintained for 3 hours to attach CECs onto the Descemet’s membrane, and (4) the corneal endothelium is ultimately regenerated by the injected CECs. The corneas of control monkeys, in which no MCECs were injected, and corneas of monkeys in which MCECs were injected without ROCK inhibitor, showed hazy corneas due to corneal endothelial dysfunction after 14 days. On the other hand, MCEC injection in combination with the ROCK inhibitor restored corneal transparency (Fig. 2a and Supplemental Fig. 1).

Scheimpflug images obtained by a Pentacam^TM^ instrument showed that an anatomically normal cornea was successfully regenerated by MCEC injection with the ROCK inhibitor, whereas corneal edema due to corneal endothelial dysfunction was induced by MCEC injection without the ROCK inhibitor (Fig. 2b). Due to severe corneal edema, Scheimpflug images were not obtained for the control eyes. Corneal thickness, which is an important index of corneal endothelial function, was significantly thinner in monkeys injected with MCECs with the ROCK inhibitor than in monkeys injected with MCECs without the ROCK inhibitor and in the control monkeys (Fig. 2c). We continued observing 2 monkeys for 1 year to evaluate the safety and efficacy of this cell-based therapy. Monkeys injected with MCECs with the ROCK inhibitor maintained corneal transparency, whereas monkeys injected only with MCECs exhibited hazy corneas after 1 year of treatment (Fig. 2d). Of the two monkeys treated with MCECs with the ROCK inhibitor, both showed a cell density higher than 2000 cell/mm^2^ (Fig. 2e). In the control eyes and eyes injected with MCECs without ROCK inhibitor, no images were obtained with non-contact specular microscopy due to corneal haziness.

The regenerated corneal endothelium had a monolayer hexagonal morphology and expressed function-related proteins, such as Na^+^/K^+^-ATPase and ZO-1 (Fig. 2f). No local adverse effects were observed, such as intraocular pressure elevation, abnormal accumulation of injected cells, or rejection. No systemic adverse responses occurred, such as abnormalities in blood tests, weight loss, or abnormal behaviors (data not shown).

### Cultivated HCEC injection in the monkey model

**Figure 3:**
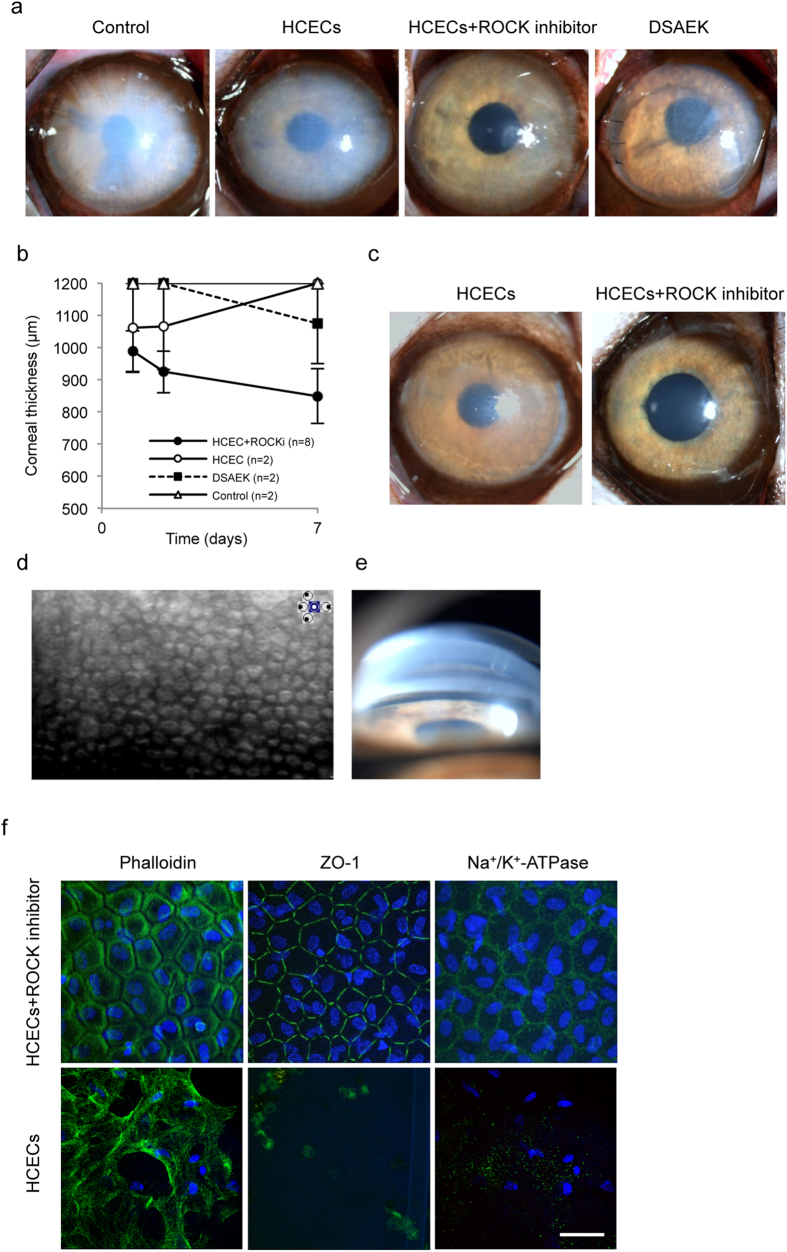
Preclinical research of cultured human corneal endothelial cell (HCEC) injection in combination with a ROCK inhibitor in a monkey corneal endothelial dysfunction model. (**a**) Representative slit-lamp images were obtained for the monkey corneal endothelial dysfunction model (n = 2). A monkey injected with HCECs (5.0 × 10^5^ cells) without the ROCK inhibitor Y-27632 (n = 2), a monkey injected with HCECs (5.0 × 10^5^ cells) and Y-27632 (n = 8), and a monkey transplanted with a human donor cornea according to DSAEK procedure (n = 2) are shown. All images were obtained 7 days after treatment. (**b**) Mean central corneal thickness was evaluated by ultrasound pachymetry. (**c**) Representative slit-lamp images were obtained for monkeys injected with HCECs without or with Y-27632. The images were obtained 3 months after injection (n = 2). (**d**) An image of the regenerated corneal endothelium was obtained by non-contact specular microscopy 3 months after treatment in a monkey injected with HCECs in combination with Y-27632. Note that no image was obtained for monkeys injected with HCECs without Y-27632. (**e**) An angle image was obtained with a gonioscopy lens 3 months after treatment of a monkey injected with HCECs in combination with Y-27632. (**f**) Cell morphology of regenerated corneal endothelium was evaluated by phalloidin staining. Function-related markers of CECs (Na^+^/K^+^-ATPase and ZO-1) showed immunostaining in the regenerated corneal endothelium. Nuclei were stained with DAPI. Scale bar: 100 μm.

We then evaluated the effect of cultured HCECs in a monkey corneal endothelial dysfunction model. The standard procedure for treating corneal endothelial dysfunction in clinical settings is Descemet’s stripping automated endothelial keratoplasty (DSAEK), so we also transplanted human donor corneas using a DSAEK procedure for comparison with the HCEC injections. As was observed with MCEC injection, HCEC injection without a ROCK inhibitor did not regenerate a transparent cornea. By contrast, HCEC injection with a ROCK inhibitor regenerated a transparent cornea by 1 week after treatment (Fig. 3a). Slitlamp microscopy revealed that corneas treated with HCECs in combination with ROCK inhibitor were as transparent as the human cornea transplanted using the DSAEK procedure (Fig. 3a). The mean corneal thickness was thinner in eyes treated with HCECs and the ROCK inhibitor than in the other two groups (Fig. 3b and Supplemental Table 1). Rejection was observed in some of the HCEC-injected eyes and DSAEK eyes due to the xeno transplantation, but the eyes injected only with HCECs and without ROCK inhibitor, where rejection did not occur, showed hazy corneas, whereas the eyes injected with HCECs in combination with the ROCK inhibitor, where rejection did not occur, retained transparent corneas for 48 days (Fig. 3c).

Specular microscopy demonstrated that eyes treated with HCECs in combination with the ROCK inhibitor had a regenerated corneal endothelium in the form of a hexagonal monolayer, with a cell density of 2890 cell/mm^2^ (Fig. 3d). Gonioscopy revealed no cell aggregation or peripheral anterior synechia. Coincidently, no intraocular pressure elevation was observed, although secondary glaucoma is a possible adverse effect of cell injection into the anterior chamber (Fig. 3e). Fluorescent staining showed that the corneal endothelium regenerated by injected HCECs was hexagonal and a monolayer, and it expressed barrier and pump function-related proteins when HCECs were injected in combination with the ROCK inhibitor. By contrast, few fibroblastic cells were observed and function-related protein expression was lost in the eyes injected only with HCECs without ROCK inhibitor (Fig. 3f).

We initiated the culture of HCECs of Good Manufacturing Practice (GMP) grade in the cell-processing center at the Kyoto Prefectural University of Medicine, using the protocol required for clinical application (Supplement Fig. 2a). We then injected GMP-grade HCECs with ROCK inhibitor to 6 monkeys as a rehearsal (a so-called cold run). Representative slitlamp and Scheimpflug images showed that the cornea became transparent and regenerated an anatomically normal cornea without corneal edema, suggesting that the HCECs cultured by the protocol for clinical application should function *in vivo* (Supplement Fig. 2b,c). Fluorescent staining also showed that the corneal endothelium regenerated by GMP-grade HCECs was hexagonal and expressed function-related proteins (Supplement Fig. 2d).

### Systemic distribution assessment

One possible adverse effect of cell-based therapy is an aberration due to the delivery of transplanted cells to other organs. We therefore evaluated the distribution of the injected HCECs in monkey organs 2 weeks after the HCEC injection. Macroscopic images and sectional analysis of organs showed no tissue abnormality and no aberrant ectopic cell transplantation (n = 6) (Supplement Figs 3 and 4). The injected HCECs were labeled with DiI fluorescence, but no DiI positive cells were observed in any organs except the corneal endothelium (data not shown).

**Figure 4:**
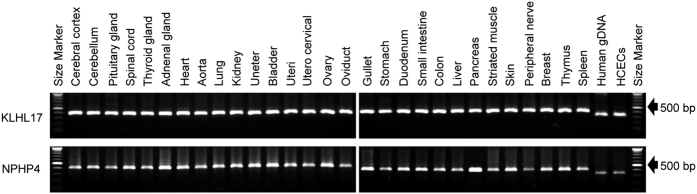
Biodistribution tests in a monkey corneal endothelial dysfunction model. PCR product sizes were compared between genomic DNAs derived from 29 different tissues from cynomolgus monkeys, and genomic DNA derived from HCECs and human genomic DNA in KLHL17 and NPHP4 genes.

We also evaluated the presence of HCECs in the corneal endothelium and other tissues using two primer sets (KLHL17 and NPHP4) that differ in the PCR product lengths in humans and cynomolgus monkeys. The 331 bp PCR products were observed in human genomic DNA and HCECs, but only the 403 bp PCR products of KLHL17 were observed in tissues of the cynomolgus monkey. Similarly, the 317 bp PCR products of NPHP4 were observed in human genomic DNA and HCECs, but the 412 bp products were observed in all tissues of the cynomolgus monkey (Fig. 4 and Supplement Fig. 5). A preliminary study was conducted to determine the detection threshold for the PCR products from human KLHL17 cells using a series of two-fold dilutions of human genomic DNA samples. The PCR products were detected using >39 pg samples of human genomic DNA as PCR templates in the first PCR amplification. The second PCR amplification was run with 1 μL of the PCR products obtained from the first PCR amplification. Hence, theoretically, if even a single human cell was included in the PCR templates, the PCR products would be detected after the second PCR amplification. These results indicate that injected HCEC-derived cells were not present in any monkey organs other than the regenerated corneal endothelium.

### A ROCK inhibitor enhances cell adhesion by suppressing the Rho/ROCK/MLC signaling cascade

**Figure 5:**
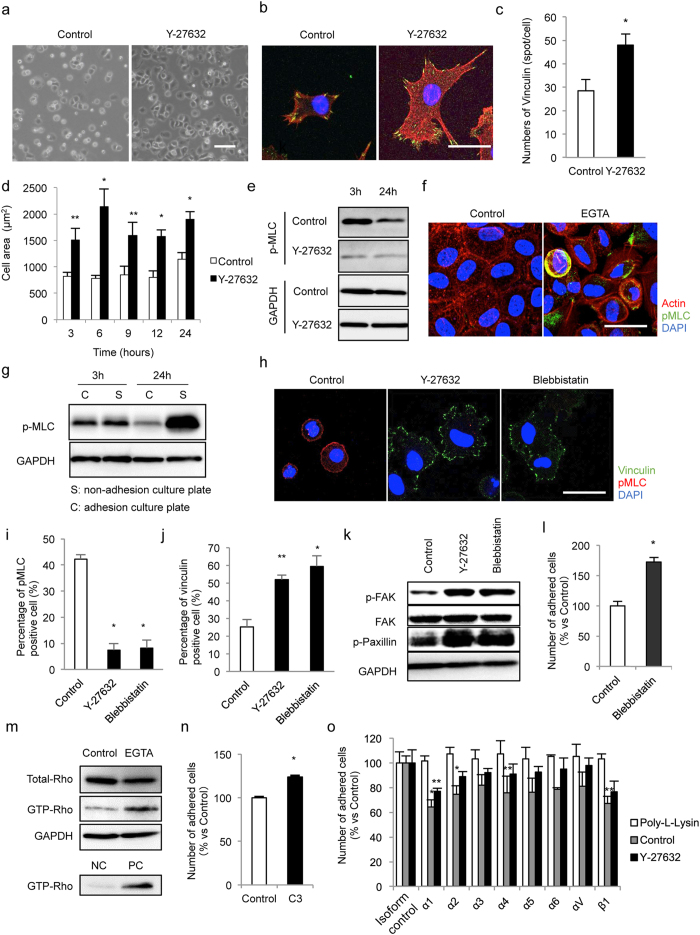
The molecular pathway by which ROCK inhibitor improves cell adhesion onto a substrate. (**a**) Phase contrast images show MCECs seeded onto culture plates after 3 hours. (**b**–**d**) MCECs seeded without or with Y-27632 were stained with phalloidin and an antibody for vinculin. The mean amount of vinculin per cell was evaluated. The mean cell area was smaller in control MCECs than in Y-27632-treated MCECs. *P < 0.01, **P < 0.05. (**e**) MLC was phosphorylated in control cells, even after 24 hours, while MLC phosphorylation was suppressed in MCECs treated with Y-27632. (**f**) Cell dissociation was induced by EGTA treatment, and phosphorylation of MLC was evaluated by immunostaining. (**g**) MCECs were seeded on non-adhesion culture plates or adhesion culture plate, and phosphorylation of MLC was evaluated by western blotting. MLC phosphorylation was sustained in MCECs seeded on non-adhesion culture plates, while it was suppressed in MCECs seeded on adhesion culture plates. (**h**–**j**) MCECs were seeded and expression of vinculin and phosphorylated MLC was evaluated by immunostaining. Control MCECs expressed phosphorylated MLC without expression of vinculin, whereas phosphorylation of MLC was suppressed in MCECs treated with a ROCK inhibitor (Y-27632) and an MLC inhibitor (blebbistatin) associated with expression of vinculin. *P < 0.01, **P < 0.05. (**k**) MCECs were seeded and the effect of inhibition of MLC on focal adhesion molecule activity was evaluated by western blotting. (**l**) MCECs were seeded with or without blebbistatin and adhered numbers of MCECs were evaluated with the CellTiter-Glo^TM^ luminescent cell viability assay after 24 hours. (**m**) Cell dissociation was induced by EGTA treatment, and activity of RhoA was evaluated by a pull-down assay. GTP-bound active RhoA was highly expressed in dissociated MCECs. (**n**) MCECs were seeded with or without C3 (a Rho inhibitor) and the numbers of adhered MCECs were evaluated by the CellTiter-Glo^TM^ luminescent cell viability assay after 24 hours. (**o**) The involvement of integrins on cell adhesion enhancement by Y-27632 was evaluated by seeding MCECs in the presence or absence of integrin-neutralizing antibodies, and the numbers of adherent cells were determined. *P < 0.01, **P < 0.05.

Phase contrast images showed that MCECs seeded without the ROCK inhibitor tended to be floating (non-adhering), with a round shape, whereas MCECs seeded with a ROCK inhibitor attached to the culture plate and showed extensive cell spreading (Fig. 5a). The actin cytoskeleton was well stretched and vinculin expression was promoted when the ROCK inhibitor was supplied with the MCECs (Fig. 5b,c). The cell size was significantly smaller in MCECs seeded without the ROCK inhibitor than with the ROCK inhibitor, even after 24 hours, suggesting that actin contraction is highly sustained by cell dissociation (Fig. 5d).

Examination of the phosphorylation of MLC showed it to be highly sustained in control MCECs and suppressed by ROCK inhibitor treatment of MCECs (Fig. 5e). Dissociation by EGTA also caused phosphorylation of MLC (Fig. 5f). The MCECs seeded on a non-adhesion plate, which maintained the cells in a dissociated state, showed phosphorylation of MLC even after 24 hours of seeding, whereas MCECs seeded on a normal culture plate exhibited less phosphorylation of MLC (Fig. 5g). These results indicate that cell dissociation induces phosphorylation of MLC and induces actin contraction in MCECs.

We also evaluated the effect of inhibiting MLC activity on adhesion of MCECs. Expression of vinculin was promoted by blebbistatin (an inhibitor of MLC) in a similar fashion to that seen in response to the ROCK inhibitor (Fig. 5h–j), as was phosphorylation of FAK and paxillin (Fig. 5k). The numbers of adhered MCECs were enhanced by blebbistatin treatment (Fig. 5l), implying that activation of MLC negatively regulates cell adhesion. When we induced cell dissociation with an EGTA treatment, the GTP-bound RhoA was highly recognized by a pull down assay, suggesting that cell dissociation induced the activation of RhoA. In turn, inhibition of RhoA activity by C3 significantly enhanced cell adhesion (Fig. 5m,n).

**Figure 6:**
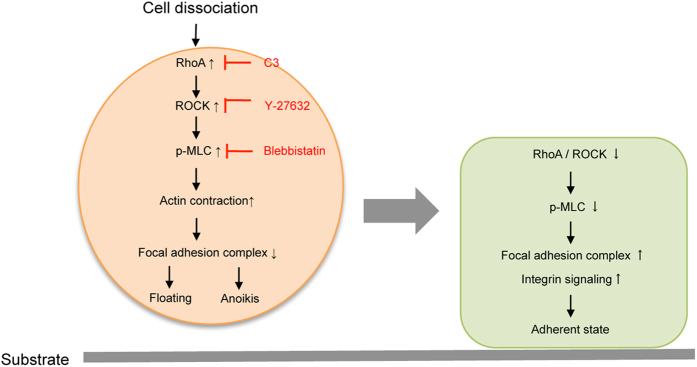
The molecular pathway by which a ROCK inhibitor treatment improves cell engraftment of CECs. (Right) Cell dissociation during the harvesting of CECs from culture plate activates RhoA/ROCK/MLC pathways. Activation of this pathway suppresses activation of the focal adhesion complex, and subsequently inhibits cell adhesion while inducing anoikis. (Left) By contrast, inhibition of actomyosin activation by a ROCK inhibitor activates the focal adhesion complex and enhances cell/ECM adhesion.

We also showed that functional blocking of integrins by neutralizing antibodies counteracted the cell adhesion enhanced by the ROCK inhibitor but not by poly-L-lysine, suggesting that the ROCK inhibitor enhances cell adhesion through interactions between the focal adhesion complex and integrins (Fig. 5o). Cell dissociation therefore appeared to upregulate a RhoA/ROCK/MLC pathway and actin contraction impeded cell adhesion. However, inhibiting the phosphorylation of MLC by a ROCK inhibitor suppressed cell shrinkage by relaxing actin contraction and then promoting the focal adhesion complex (Fig. 6).

## Discussion

The low efficiency of engraftment and loss of phenotype after transplantation due to the absence of cell/cell and cell/ECM interactions *in vivo* impairs organ reconstruction in various tissues^9^,^10^,^11^. Researchers have therefore been exploring the use of various techniques, such as artificial scaffolds, biologically active molecules, and ECM coatings, to improve cell retention and survival^12^,^13^,^14^. In the present study, we defined the inhibition of ROCK signaling as a novel target for improving engraftment in the setting of cell-based therapy. We showed that CECs recovered from a culture plate for cell therapy undergo dissociation-induced Rho/ROCK/MLC signaling activation, which then impairs cell engraftment. Inhibition of ROCK therefore enhances cell engraftment and maintains the phenotype of the transplanted cells. Similar to our findings, Ohgushi and colleagues reported that human embryonic stem cells are vulnerable to apoptosis following dissociation due to ROCK-dependent hyperactivation of actomyosin, and that Rho-GEF (guanine nucleotide exchange factor), containing a functional Rac-GAP (GTPase activating protein) domain, is an indispensable regulator of Rho/ROCK/myosin activation^15^. The dissociation-induced cellular response varies with the cell type, but our findings should encourage researchers to evaluate and modulate dissociation-induced Rho/ROCK activation to improve cell engraftment efficiency in the settings of cell-based therapies in other organs.

We previously demonstrated that a ROCK inhibitor promotes the adhesion of cultured MCECs^16^, and a subsequent report by another group has also confirmed that a ROCK inhibitor improved the attachment of HCECs^17^. In 2012, we showed that use of ROCK inhibitor enables efficient cell engraftment in rabbit corneal endothelial dysfunction models and in a very preliminarily monkey model^8^. However, the rabbit corneal endothelium has a proliferative ability that is lacking in humans^18^,^19^, leaving the possibility that the proliferation of injected rabbit CECs seen after transplantation and the subsequent reconstruction of the corneal endothelium, would not be expected to occur in humans. We employed the present primate model to confirm or eliminate this possibility, because this model resembles the human corneal endothelium in terms of its very limited proliferative potency^18^,^19^. The use of this monkey model demonstrated that injection of either monkey or human CECs in combination with a ROCK inhibitor reconstructed the monkey corneal endothelium.

Corneal endothelial dysfunction has been treated by full-thickness corneal transplantation (penetrating keratoplasty) for more than 90 years, but more selective corneal endothelial replacement such as Descemet’s stripping endothelial keratoplasty (DSEK) and Descemet’s membrane endothelial keratoplasty (DMEK) were developed in the last decade^3^,^20^. In addition, graft rejection rates during first 2 years after corneal transplantation were 1% in DMEK and 5–14% in DSEK^20^,^21^,^22^,^23^,^24^,^25^,^26^,^27^ suggesting that simple replacement of the corneal endothelium induce fewer episodes of rejection and that the stroma, but not corneal endothelium, mainly triggers antigenic recognition and responses^20^. The clinically successful outcomes of DSEK and DMEK imply that reconstruction of the corneal endothelium is a definitive treatment that can replace full thickness corneal replacement, and that further corneal endothelium reconstruction by cell-based therapy is a clinically relevant approach.

At present, HCECs are isolated from donor corneas and cultured for research purposes^28^, but several substantial technical obstacles remain, such as limited proliferative ability, vulnerable transformation with loss of functions, and senescence that prohibits efficient *in vitro* expansion for clinical use^16^,^29^,^30^,^31^,^32^,^33^,^34^. Indeed, no protocol specifically designed for clinical application has been established, although our research group and others are continually striving towards the development of a successful culture method^29^,^30^,^31^,^34^. For instance, we reported that a conditioned medium obtained from GMP-grade human bone marrow-derived mesenchymal stem cells (BM-MSCs) enhanced CEC proliferation^33^ and that inhibition of transforming growth factor beta (TGF-β) signaling activation by small molecules maintains the functional phenotype by counteracting fibroblastic transformation^32^. Based on these findings, we are currently culturing HCECs of Good Manufacturing Practice (GMP) grade in the cell-processing center for use in clinical applications^35^.

In the United States, the U.S. Food and Drug Administration (FDA) regulates regenerative medicine products through the Center for Biologics Evaluation and Research (CBER) and other countries also have similar systems^36^. Working with the FDA requirements^11^, we tested the safety and efficacy of GMP-grade HCECs in the same fashion as we intend to use in the transplantation of these cells into human patients in clinical trials. We have now established a culture protocol for clinical use. Notably, based on this current preclinical research, in 2013, we initiated a first-in-man clinical trial of cell-based therapy to treat corneal endothelial dysfunction at the Kyoto Prefectural University of Medicine, after obtaining the necessary approval (Clinical trial registration: UMIN000012534) from the Japanese Ministry of Health, Labour and Welfare.

The major safety concern of any regenerative medicine product is the potential for tumor formation^36^. There is a spectrum of risk (e.g., differentiated somatic cells have a lower risk than undifferentiated embryonic stem cells), so product-specific evaluation is necessary as a preclinical testing strategy^36^. In the current study, comparison of the numbers of injected CECs and the cell density of the regenerated corneal endothelium revealed that 40–50% of the injected CECs adhered to the cornea. The residual cells were then considered to have the potential to flow out by aqueous flow to other organs via the veins. We therefore examined the distribution of transplanted CECs in multiple organs of monkey disease models and showed that no CECs were observed by fluorescein labeling tracing and PCR at 2 weeks after transplantation (although perfect *in vivo* monitoring of delivered cells is surprisingly challenging). One possible explanation is that any cells that flowed out were removed by the host immune system; however, the duration of the follow-up period used here was not sufficient to conclude that these residual cells do not hold any risk of tumorigenicity. Therefore, diligent feedback from clinical trials—by evaluating possible side effects, such as host immune response and pulmonary embolism—will be needed to ensure patient safety and protect vulnerable populations.

In conclusion, inhibiting the dissociation-induced actomyosin activation by a ROCK inhibitor improves the efficiency of engraftment of CECS and enables cell-based approaches for treating corneal endothelial dysfunction as a clinically relevant therapy.

## Methods

### Ethics statement

Animals were housed and treated in accordance with the ARVO Statement for the Use of Animals in Ophthalmic and Vision Research. The monkey experiments were performed at the Research Center for Animal Life Science at Shiga University of Medical Science (Otsu, Japan) according to the protocol approved by that university’s Animal Care and Use Committee (Approval No. 2012-1-6H). Human donor corneas were obtained from SightLife^TM^ (http://www.sightlife.org/, Seattle, WA) for research purposes.

### Cell Culture

Ten corneas from 5 cynomolgus monkeys (3 to 5 years-of-age; estimated equivalent human age: 5 to 20 years) housed at NISSEI BILIS Co., Ltd. and Eve Bioscience, Co., Ltd. were used for the MCEC culture. The MCECs were cultivated as described previously^7^. Briefly, the Descemet’s membrane with MCECs was stripped and incubated in 1 mg/mL collagenase A (Roche Applied Science, Penzberg, Germany). The isolated MCECs were resuspended in culture medium and seeded on culture plates coated with FNC Coating Mix^®^ (Athena Environmental Sciences, Inc., Baltimore, MD). All primary cell cultures and serial passages of MCECs were performed in a growth medium composed of Dulbecco’s modified Eagle’s medium (Life Technologies Corp., Carlsbad, CA) supplemented with 10% fetal bovine serum (FBS), 50 U/mL penicillin, 50 μg/mL streptomycin, and 2 ng/mL fibroblast growth factor 2 (Life Technologies Corp.). MCECs at passages 2 through 8 were used for these experiments.

A total of ten human donor corneas were used for cultivation of HCECs by the protocol described previously^32^,^33^. Briefly, the Descemet’s membranes containing the HCECs were stripped, followed by digestion with 1 mg/mL collagenase A for 12 hours. The HCECs were seeded and cultured in HCEC culture medium prepared according to published protocols. Basal medium, composed of OptiMEM-I (Life Technologies Corp.), 8% FBS, 5 ng/mL epidermal growth factor (Sigma-Aldrich Co., St. Louis, MO), 20 μg/mL ascorbic acid (Sigma-Aldrich Co.), 200 mg/L calcium chloride, 0.08% chondroitin sulfate (Wako Pure Chemical Industries, Ltd., Osaka, Japan), 50 μg/mL gentamicin, and 10 μM SB431542 (Merck Millipore, Billerica, MA), was conditioned by culturing human bone marrow-derived mesenchymal stem cells (BM-MSCs) for 24 hours. The basal medium conditioned with BM-MSCs was collected for use as the culture medium for HCECs. HCECs at passages 2 through 5 were used for these experiments.

### Injection of CECs into a monkey corneal endothelial dysfunction model

The monkey corneal endothelial dysfunction model was created by scraping the corneal endothelium completely from the Descemet’s membrane with a 20-gauge silicone needle (Soft Tapered Needle; Inami & Co., Ltd., Tokyo, Japan) while the animal was under general anesthesia, as described previously^8^. The MCEC injection experiments were conducted on the following 3 groups: 1) MCECs (5.0 × 10^5^ cells) were suspended in 200 μl of DMEM supplemented with 100 μM of Y-27632 (ROCK inhibitor; Wako Pure Chemical Industries, Ltd.) and injected into the anterior chamber (n = 6), (2) MCECs (5.0 × 10^5^ cells) were suspended in 200 μl of DMEM and injected into the anterior chamber (n = 2), and (3) no MCECs were injected (n = 2). The HCEC injection experiments were conducted on the following 4 groups:1) HCECs (5.0 × 10^5^ cells) were suspended in 200 μl of OptiMEM-I supplemented with 100 μM of Y-27632 (Wako Pure Chemical Industries, Ltd.) and injected into the anterior chamber (n = 8), (2) HCECs (5.0 × 10^5^ cells) were suspended in 200 μl of OptiMEM-I and injected into the anterior chamber (n = 2), (3) human pre-cut donor corneas were transplant using a DSAEK procedure (n = 2), and (4) no HCECs were injected(n = 2). The eyes were kept in the face-down position for 3 hours under general anesthesia, except for the eyes of monkeys that underwent DSAEK. The corneal transparency and thickness of the anterior segments were evaluated by slitlamp microscopy. A Pentacam^®^ (OCULUS Optikgeräte GmbH, Wetzlar, Germany) instrument was used to visualize the corneal shape. Corneal thickness was determined with an ultrasound pachymeter (SP-2000; Tomey, Nagoya, Japan), and the mean of 10 measured values was calculated (up to a maximum thickness of 1200 μm, the instrument’s maximum reading). Intraocular pressure was determined by a Tonovet^®^ (icare Finland, Vantaa, Finland) instrument. The corneal endothelium was evaluated by non-contact specular microscopy (FA-3809, Konan Medical, Nishinomiya, Japan). Eyes that exhibited clinical features such as the presence of keratic precipitates, progression of corneal edema, and conjunctival injection were diagnosed as having undergone graft rejection.

### Immunohistochemistry

Samples were fixed for 20 min with 4% paraformaldehyde and excess paraformaldehyde was removed by washing with Dulbecco’s phosphate-buffered saline (PBS). The samples were permeabilized with 0.3% Triton^**®**^X-100 (Nacalai Tesque, Kyoto, Japan), and then incubated with 1% bovine serum albumin (BSA) to block nonspecific binding. Specimens were incubated with primary antibodies against Na^+^/K^+^-ATPase (1:300, Upstate Biotechnology, Lake Placid, NY), ZO-1 (1:300, Life Technologies Corp.), and N-cadherin (1:300, BD Biosciences, San Jose, CA), connexin 43 (1:300, Life Technologies Corp.), and vinculin (Merck Millipore, Billerica, MA). Alexa Fluor^®^ 488- or 594- conjugated goat anti-mouse (Life Technologies Corp.) antibodies were used as secondary antibodies at a 1:1000 dilution. Actin staining was performed by incubation with a 1:400 dilution of Alexa Fluor^®^ 488- or 546-conjugated Phalloidin (Life Technologies Corp.). Nuclei were stained with DAPI (Vector Laboratories, Burlingame, CA). The slides were examined with a fluorescence microscope (TCS SP2 AOBS; Leica Microsystems, Wetzlar, Germany).

### Immunoblotting

The MCECs were washed with ice-cold PBS, lysed with ice-cold RIPA buffer containing phosphatase inhibitor cocktail 2 (Sigma-Aldrich Co., St. Louis, MO) and protease inhibitor cocktail (Roche Applied Science, Penzberg, Germany), and then centrifuged. The supernatant representing total proteins was collected and fractionated by SDS-PAGE. The proteins were then transferred to PVDF membranes and blocked with 3% non-fat dry milk, followed by an overnight incubation at 4 °C with the following primary antibodies: phosphorylated focal adhesion kinase (FAK) (1:1000; Cell Signaling Technology, Inc., Danvers, MA), FAK (1:1000; Cell Signaling Technology), phosphorylated MLC (Merck Millipore, Billerica, MA), phosphorylated paxillin (1:1000; Cell Signaling Technology), and GAPDH (1:3000; Abcam, Cambridge, UK). The blots were probed with horseradish peroxidase-conjugated secondary antibodies (1:5000; Cell Signaling Technology), followed by development with luminal for enhanced chemiluminescence using the ECL Advanced Western Blotting Detection Kit (GE Healthcare, Piscataway, NJ), and documentation by an LAS4000S (Fuji Film, Tokyo, Japan) cooled charge-coupled-device camera gel documentation system. Molecular weight markers (Bio-Rad, California) were run alongside all samples. The relative density of the immunoblot bands was determined by Image J^®^ (NIH) software.

### Rho pull down assay

The RhoA activation was evaluated by a Rho activation assay (Merck Millipore, Billerica, MA) according to the manufacturer’s protocol. MCECs were cultured to a confluent state, and dissociated by incubation in serum-free medium supplemented with 3 mM EGTA(Nacalai Tesque) for 16 hours. The MCECs were washed with ice-cold PBS, lysed with ice-cold Mg2^+^ Lysis/Wash Buffer (Merck Millipore) containing phosphatase inhibitor cocktail 2 (Sigma-Aldrich Co.), and then agitated. Samples were then reacted with Rho Assay Reagent (Merck Millipore) to bond GTP-Rho. The supernatant representing total proteins was collected and immunoblotting was performed described as above. Anti-Rho, clone 55, (3:1000; Merck Millipore) was used as the primary antibody.

### Cell adhesion assay

The involvement of integrins in the enhancement of cell adhesion by the ROCK inhibitor was evaluated by seeding MCECs (5 × 10^3^ cells/well) in 96-well plates in the presence or absence of integrin-neutralizing antibodies (2 μg/mL): anti-α1 integrin (Merck Millipore, Billerica, MA), anti-α2 integrin (Merck Millipore, Billerica, MA), anti-α3 integrin (Merck Millipore, Billerica, MA), anti-α4 integrin (Merck Millipore, Billerica, MA), anti-α5 integrin (Merck Millipore, Billerica, MA), anti-α6 integrin (Merck Millipore, Billerica, MA), anti-αV integrin (Merck Millipore, Billerica, MA), anti-α6 integrin (Merck Millipore), and anti-β1 integrin (R&D systems Inc., Minneapolis, MN). The effect of inhibiting phosphorylation of MLC and RhoA activity on cell adhesion was also evaluated by seeding MCECs with blebbistatin (10 μM) and C3 (300 ng/ml), respectively. Three hours after seeding, the numbers of adherent cells were determined with the CellTiter-Glo^TM^ luminescent cell viability assay (Promega Corporation, Madison, WI) according to the manufacturer’s instructions. The number of adhered cells was determined using a Veritas^TM^ microplate luminometer (Promega Corporation).

### PCR method

Genomic DNAs from cynomolgus monkey tissues were extracted using the DNeasy Blood & Tissue kit (Qiagen, Hilden, Germany), and the quality was measured with a NanoDrop^®^ spectrophotometer (Thermo Fisher Scientific Inc., Waltham, MA). The KLHL17 and NPHP4 genes, which differ in length in humans and cynomolgus monkeys, were selected by a detailed search between human and rhesus monkey reference sequences (GRCh37/hg19 and MGSC Merged 1.0/rheMac2) released in the UCSC website (http://genome.ucsc.edu/index.html) for designation of primers. Two new primer sets in KLHL17 and NPHP4 genes were designed in exon 7 and exon 8 (PCR product sizes: 331 bp in human and 403 bp in cynomolgus monkey) and intron 11 (PCR product sizes: 317 bp in human and 412 bp in cynomolgus monkey), respectively, with the following primers: KLHL17 (KLHL17_F1: 5′- TGGTGGCCTCCATGTCCAC-3′ and KLHL17_R1: 5′- CTACCTGTTCAGGCAGGAG-3′), NPHP4 (NPHP4_F1: 5′- GGTGCTTCCCAAACTATACT-3′ and NPHP4_R1: 5′- GGTAGCTTCCATTTGCAGGA-3′). The fixing of the PCR products sizes in humans was confirmed by the 1000 genome website (http://www.1000genomes.org). In brief, the 20 μL amplification reaction volume contained 20 ng of genomic DNA, 0.4 units of KOD FX polymerase (TOYOBO, Osaka, Japan), 2 × PCR buffer, 2 mM of each dNTP and 0.5 μM of each primer. The cycling parameters were as follows: an initial denaturation of 94 °C/2 min followed by 30 cycles of 98 °C/10 sec, 62 °C/15 sec and 68 °C/30 sec. The irreducible minimum HCECs were detected by performing two repeats of the PCR amplification process. The second PCR amplification was run with 1 μL of PCR products obtained from the first PCR amplification. The PCR reactions were performed using the thermal cycler GeneAmp PCR system 9700 (Applied Biosystems/Life Technologies/Thermo Fisher Scientific, Foster City, CA). The PCR products were separated by electrophoresis on 1.0% agarose gels, stained with ethidium bromide, and detected under ultraviolet illumination.

### Statistical analysis

The statistical significance (P-value) of differences between mean values of the two-sample comparison was determined with the Student’s t-test. The comparison of multiple sample sets was analyzed using Dunnett’s multiple-comparison test. The values shown in the graphs represent the mean ± SEM.

## Additional Information

**How to cite this article**: Okumura, N. *et al.* Rho kinase inhibitor enables cell-based therapy for corneal endothelial dysfunction. *Sci. Rep.* **6**, 26113; doi: 10.1038/srep26113 (2016).

## Supplementary Material

Supplementary Information
