# Supplementary material for: Rho kinase inhibitor enables cell-based therapy for corneal endothelial dysfunction
Source: Sci Rep. 2016 May 18;6:26113. doi: 10.1038/srep26113 (PMC4870691; doi:10.1038/srep26113)
Supplement: Supplementary Information [file srep26113-s1.pdf]

Supplementary information

Rho kinase inhibitor enables cell-based therapy for corneal endothelial dysfunction

Naoki Okumura[1], Yuji Sakamoto[2], Keita Fujii[1], Junji Kitano[1], Shinichiro Nakano[1], Yuki Tsujimoto[1], Shin-ichiro Nakamura[3], Morio Ueno[4], Michio Hagiya[4], Junji Hamuro[4], Akifumi Matsuyama[5], Shingo Suzuki[6], Takashi Shiina[6], Shigeru Kinoshita[4,7], and Noriko Koizumi[1]

[1]Department of Biomedical Engineering, Faculty of Life and Medical Sciences, Doshisha University, Kyotanabe, Japan
[2]Research Laboratory, Senju Pharmaceutical Co., Ltd., Kobe, Japan
[3]Research Center of Animal Life Science, Shiga University of Medical Science, Otsu, Japan
[4]Department of Ophthalmology, Kyoto Prefectural University of Medicine, Kyoto, Japan
[5]Platform of Therapeutics for Rare Disease, National Institutes of Biomedical Innovation, Health and Nutrition, Osaka, Japan
[6]Department of Molecular Life Science, Division of Basic Medical Science and Molecular Medicine, Tokai University School of Medicine, Isehara, Japan
[7]Department of Frontier Medical Science and Technology for Ophthalmology, Kyoto Prefectural University of Medicine, Kyoto, Japan

Corresponding author
Noriko Koizumi, M.D., Ph.D., Department of Biomedical Engineering, Faculty of Life and Medical Sciences, Doshisha University, Kyotanabe 610-0321, Japan.
E-mail: nkoizumi@mail.doshisha.ac.jp
Tel and Fax: +81-774-65-6125

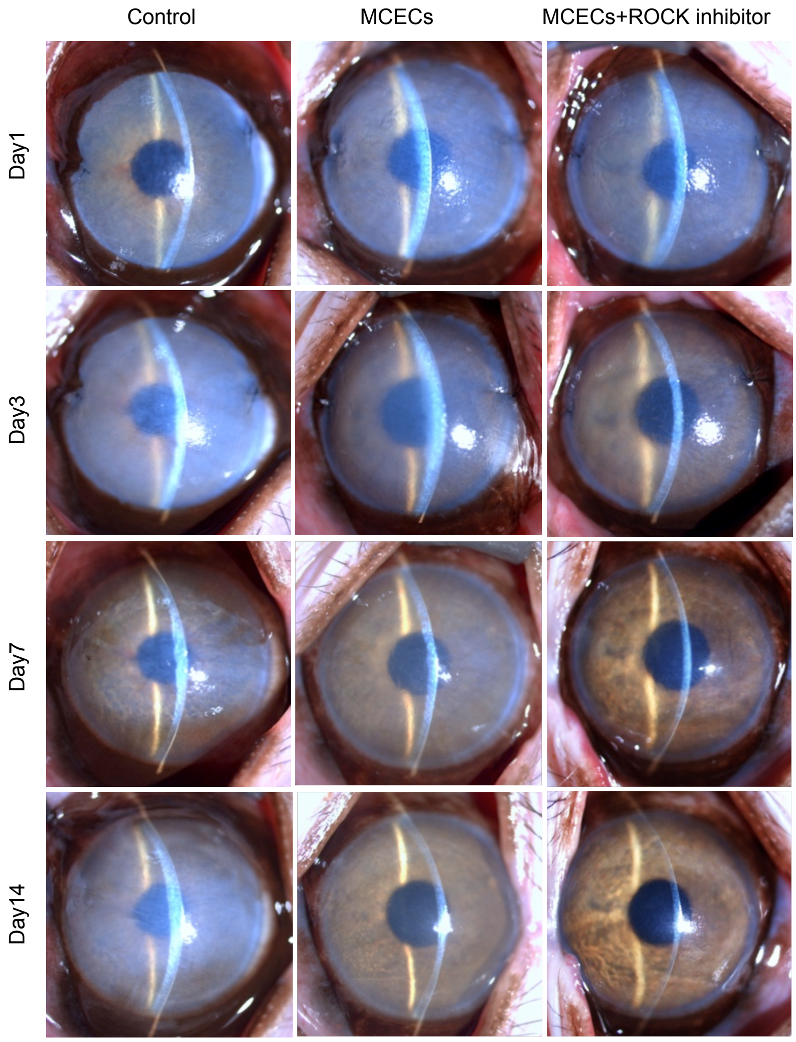

Supplemental Figure 1. Cultured monkey corneal endothelial cell (MCEC) injection in combination with a ROCK inhibitor in a monkey corneal endothelial dysfunction model.

A slit-lamp image shows the monkey corneal endothelial dysfunction model (left) (n=2). A slit-lamp image shows the corneal endothelial dysfunction model following injection of MCECs (5.0×10^5 cells) suspended in 200 µl of DMEM without (middle) (n=2) or with (right) (n=6) the ROCK inhibitor Y-27632.

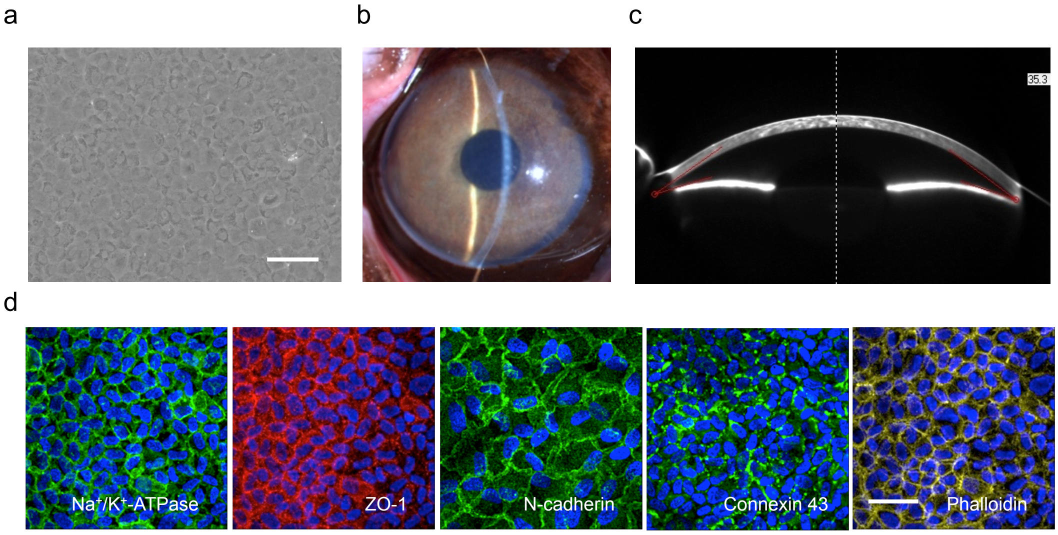

Supplemental Figure 2. Injection of GMP-grade cultured HCECs in a monkey corneal endothelial dysfunction model.

(a) For evaluation of the HCECs cultured according to the protocol for clinical application, HCECs were cultured as Good Manufacturing Practice (GMP) grade in the cell-processing center at the Kyoto Prefectural University of Medicine (Kyoto, Japan). A phase contrast image was obtained for HCECs of Good Manufacturing Practice GMP grade cultured in the cell-processing center.

(b) The safety and efficacy of GMP-grade HCECs was the same as those planned for transplantation to human patients in clinical trials and were evaluated in a monkey disease model. The resulting HCECs (5.0×10^5 cells) were suspended in 200 µl of OptiMEM-I supplemented with 100 µM of Y-27632 and injected into the anterior chamber (n=6). A representative slit-lamp image was obtained for the monkey disease model injected with HCECs in combination withY-27632. The image was obtained 7 days after injection.

(c) Scheimpflug images were obtained for a monkey injected with HCECs planned for transplantation to human patients. The images were obtained with a Pentacam® instrument 7 days after cell injection.

(d) Cell morphology of regenerated corneal endothelium was evaluated by phalloidin staining. Function-related markers of CECs (Na^+/K^+-ATPase, ZO-1, N-cadherin, and connexin 43) showed immunostaining in the regenerated corneal endothelium. Nuclei were stained with DAPI. Scale bar: 100 µm.

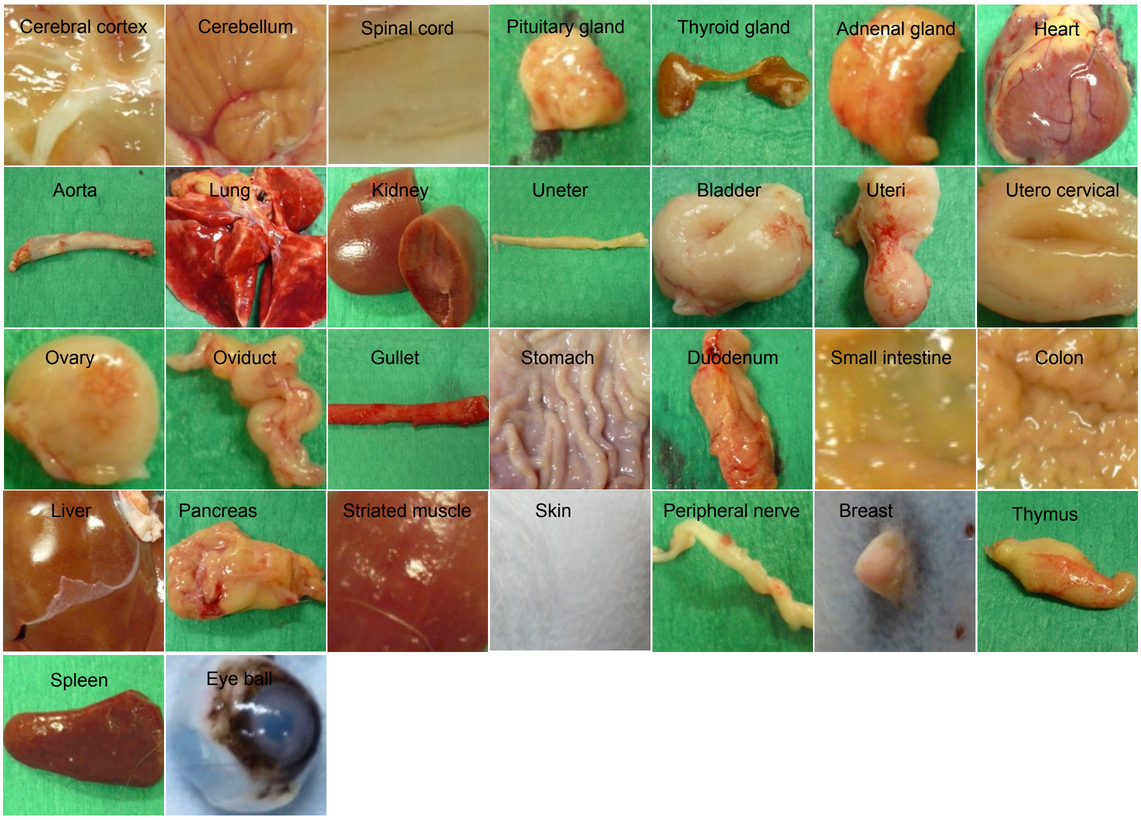

Supplemental Figure 3. Biodistribution tests in monkey model (macroscopic analysis). Macroscopic images were obtained of monkey organs.

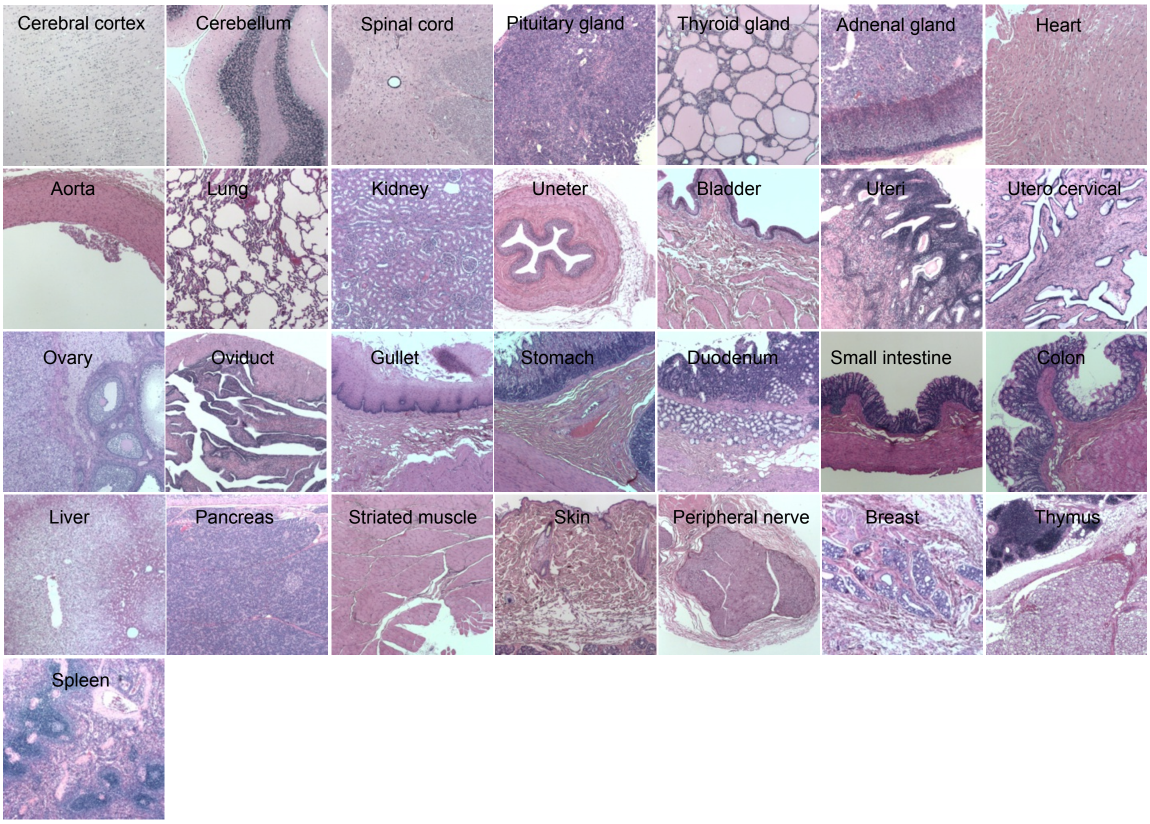

Supplemental Figure 4. Biodistribution tests in monkey model (microscopic analysis). Hematoxylin and eosin staining images were obtained for different monkey organs.

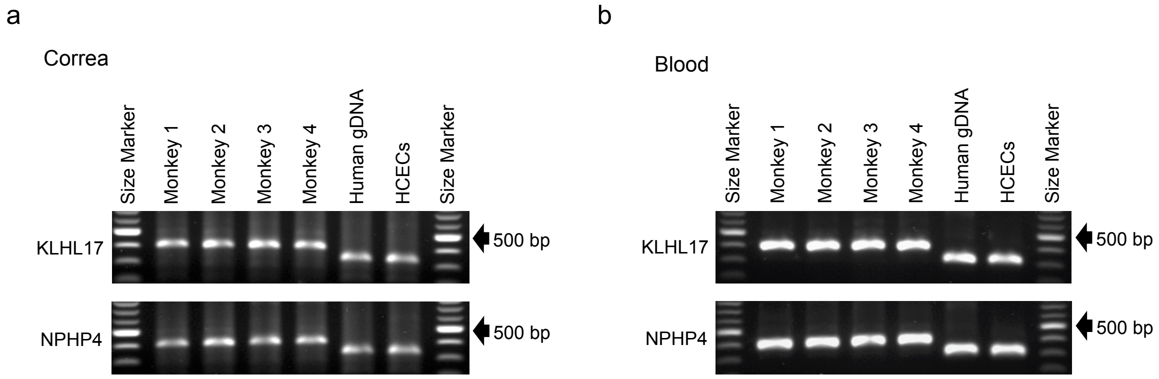

Supplemental Figure 5. Biodistribution tests in monkey model (PCR analysis).
(a) The KLHL17 and NPHP4 gene PCR product sizes were compared between genomic DNAs derived from corneal endothelium of 4 independent cynomolgus monkeys, human genomic DNAs, and cultured human corneal endothelium cells (HCECs).
(b) The KLHL17 and NPHP4 gene PCR product sizes were compared between genomic DNAs derived from peripheral blood cells of 4 independent cynomolgus monkeys, human genomic DNAs, and cultured HCECs.

Time (day)	1	2	7
HCEC+ROCKi (No.1)	1200	1200	523
HCEC+ROCKi (No.2)	813	704	684
HCEC+ROCKi (No.3)	1200	881	725
HCEC+ROCKi (No.4)	877	836	773
HCEC+ROCKi (No.5)	797	838	804
HCEC+ROCKi (No.6)	884	790	880
HCEC+ROCKi (No.7)	944	947	1200
HCEC+ROCKi (No.8)	1200	1200	1200
HCEC (No.1)	1200	1200	1200
HCEC (No.2)	923	932	1200
DSAEK (No.1)	1200	1200	949
DSAEK (No.2)	1200	1200	1200
Control (No.1)	1200	1200	1200
Control (No.2)	1200	1200	1200

Values were shown in µm.
HCEC+ROCKi (No.1, 2, 4, 5, 6, and 8) and DSAEK (No.1) showed graft rejection on day 7-14.

Supplemental Table 1. Central corneal thickness of monkey corneal endothelial dysfunction model injected with cultured human corneal endothelial cell (HCEC).
